# Structure, function, and productivity from the National Dental Practice-Based Research Network

**DOI:** 10.1017/cts.2022.421

**Published:** 2022-06-22

**Authors:** Gregg H. Gilbert, Jeffrey L. Fellows, Veerasathpurush Allareddy, David L. Cochran, Joana Cunha-Cruz, Valeria V. Gordan, Mary Ann McBurnie, Cyril Meyerowitz, Rahma Mungia, D. Brad Rindal, National Dental PBRN Collaborative Group

**Affiliations:** 1 Department of Clinical and Community Sciences, School of Dentistry, University of Alabama at Birmingham, Birmingham, AL, USA; 2 Kaiser Permanente Center for Health Research, Portland, OR, USA; 3 Department of Orthodontics, College of Dentistry, University of Illinois at Chicago, Chicago, IL, USA; 4 Department of Periodontics, School of Dentistry, University of Texas Health Science Center at San Antonio, San Antonio, TX, USA; 5 Department of Restorative Dental Sciences, College of Dentistry, University of Florida, Gainesville, FL, USA; 6 Eastman Institute for Oral Health, Rochester, NY, USA; 7 HealthPartners Institute for Education and Research, Minneapolis, MN, USA; 8 The National Dental PBRN Collaborative Group comprises practitioners, faculty, and staff investigators who contributed to this network activity, listed at http://www.nationaldentalpbrn.org/collaborative-group.php

**Keywords:** Research infrastructure, multicenter studies, metrics, practice-based research networks, practice patterns

## Abstract

**Introduction:**

Following inception in 2005 as a multiregional practice-based research network (PBRN), the “National Dental PBRN” expanded nationwide in 2012, and in 2019 implemented additional organizational changes. The objectives are to: (1) describe the new structure and function of the network; and (2) quantify its scientific productivity since 2005.

**Methods:**

A national Administrative and Resource Center is based in Alabama; regional and specialty nodes are based in Alabama, Florida, Illinois, Minnesota, Oregon, New York, and Texas. A Network Coordinating Center is based in Oregon. Studies are funded via investigator-initiated grants. Scientific productivity is assessed using specific metrics, including the Relative Citation Ratio.

**Results:**

To date, 58 studies have been completed or are in data collection or development. These studies have investigated a broad range of topics using a wide variety of study designs. Of the studies that have completed enrollment, 70,665 patients were enrolled, as were 19,827 practitioners (some participated in multiple studies), plus electronic records for 790,493 patients in two data-only studies. To date, these studies have led to 193 peer-reviewed scientific publications in 62 different journals. The mean (1.40) Relative Citation Ratio of Network publications connotes a greater-than-average influence in their fields.

**Conclusions:**

These metrics demonstrate that the PBRN research context can successfully engage practitioners and patients from diverse settings nationally with a high and sustained level of scientific productivity. This infrastructure has enabled clinical scientists in oral health and nonoral health topics and provided additional recruitment venues outside of the typical academic health center research context.

## Introduction

Practice-based research networks (PBRNs) offer unique advantages to clinical research and quality improvement [1-6], primarily because they bring practice-relevant topics onto the research agenda and are conducted in “real-world,” nonacademic clinical settings where almost all of the population receives its health care. Historically most PBRNs have focused on medical care [7], but a recent review documented the growth in number of dental PBRNs [8]. This review concluded that the largest dental PBRN globally is the “National Dental PBRN” (“Network”), and relied on the Network’s most-recent publication about these topics (from 2013) [9]. From 2005 to 2012, the Network operated as a multiregional PBRN in four USA regions and one region that comprised three Scandinavian countries. From 2012 to 2019, the Network became nationwide throughout the USA as six regions and no longer operated a Scandinavian region. Beginning in 2019, new organizational changes were made, the description of which comprises one purpose of this current article.

Journal impact factor and the h-index [10] are commonly used measures of scientific impact. However, these metrics have important limitations, such as obscuring large differences in the influence of individual articles or undervaluing some fields of research by failing to normalize raw citation counts. In an effort to address the limitations of these and other measures, the Relative Citation Ratio (RCR) was developed to quantify the influence of a research article that is article level and independent of the scientific field [11]. To facilitate its public use, a National Institutes of Health (NIH) PubMed site was established [12]. All peer-reviewed articles from NIH-funded studies are required to be publicly available in this database [13].

The aforementioned review [8] compared scientific productivity between PBRNs and independent investigative teams. These comparisons included number of publications, range of clinical topics studied, and number of practitioners and patients enrolled. Only limited information was available to the authors about network-specific productivity. Indeed, reports of PBRN performance metrics are rare for both medical and dental PBRNs, and have primarily focused on the number of studies completed and the number of participants involved [6,14,15]. There is no broad consensus about which metrics to quantify productivity across PBRNs and other research networks. The National Dental PBRN has reported productivity based on the metrics of practitioner engagement (e.g., practitioner participation in studies, webinars, network activities that provide continuing education credit, presentations and publications, and practitioner meetings) [16,17], but has not reported other measures of overall network scientific productivity, such as the number of peer-reviewed publications and impact as measured by RCR. Therefore, this article aims to: (1) describe the new organizational structure and function of the Network and (2) quantify the Network’s scientific productivity.

## Methods

The Network’s mission is “To improve oral health by conducting dental practice-based research and by serving dental professionals through education and collegiality.” It seeks to maximize the practicality of conducting research in everyday clinical practice across geographically dispersed regions and diverse practice types. Its structure is designed to focus some activities at the regional level (e.g., interactions with practitioners), while managing other activities centrally (e.g., study development).

### Overall Network Structure and Oversight

**Fig. 1.**
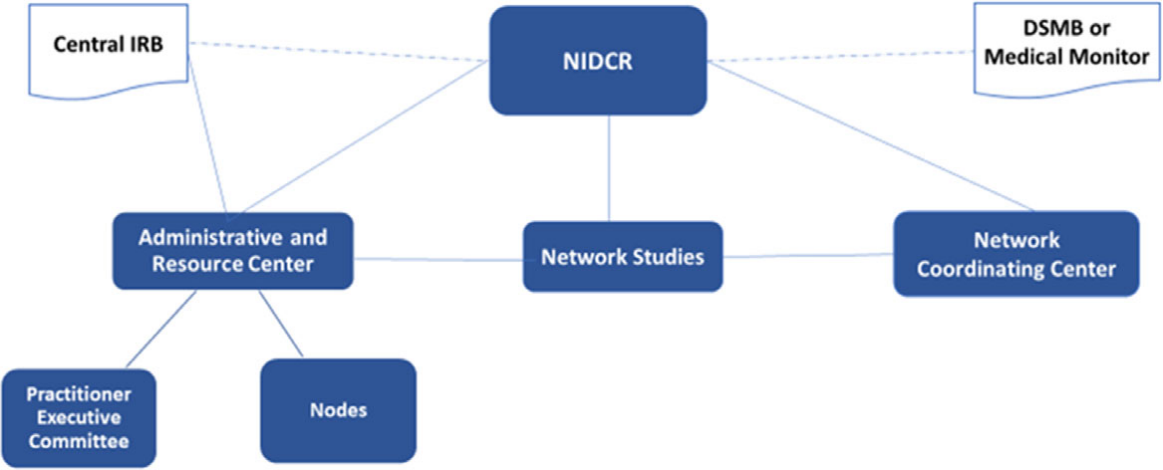
**Overall network structure and oversight.** DSMB: Data Safety and Monitoring Board; IRB: Institutional Review Board; NIDCR: National Institute of Dental and Craniofacial Research

The overall structure of the Network was revised in 2019 [18,19], as depicted in Fig. 1. The Network’s main funder is NIH. An Administrative and Resource Center (ARC) and Network Coordinating Center (NCC) support the infrastructure for study development and implementation. The NCC is located in Oregon and provides both scientific and administrative functions. NCC biostatisticians support study development and analysis plans and NCC management staff provide support for study operations and data management. The NCC also designs and implements technology for the network “Hub” which supports the practitioner and participation databases; houses key network-wide documents; implements and tracks study data collection, data quality management, study monitoring procedures, and data analysis. The ARC is the national administrative base (located in Alabama) for six regional nodes and one specialty node that span all 50 US states and territories. Nodes are administratively based in Alabama, Florida, Minnesota, New York, Oregon, Illinois, and Texas. All enrolled practitioners are associated with one of these regional centers. The ARC also directs “Components” focused on specific administrative tasks: a Communications and Dissemination Component; a Practitioner Recruitment and Engagement Component; a Practitioner Training Component; and a Practitioner and Patient Compensation System, which are based either in Alabama or Florida. Each Component Director and Node Director reports to the National Network Director, who is responsible for overall scientific, technical, and administrative leadership and who has primary responsibility for planning and directing Network infrastructure and managing Network operations and fiscal resources. Also shown in Fig. 1, the ARC manages the Practitioner Executive Committee (PEC) and Network interactions with the Central Institutional Review Board (CIRB; ethics committee). The PEC comprises practitioner representatives from each Network region, who provides input about the design, feasibility, and clinical interest of studies and research topics. The Network’s CIRB has been in operation since 2014 and enables the Network to comply with the NIH policy that requires a single IRB review for multi-site studies involving nonexempt human subject research in which each site conducts the same protocol.

Fig. 1 also depicts a Data and Safety Monitoring Board (DSMB). The DSMB is an independent group of experts that advises the NIH and study investigators on clinical studies, especially studies that involve an intervention. Its responsibilities include monitoring human subject safety; evaluating study data; reviewing study conduct and progress; and making recommendations to NIH concerning a study’s continuation, modification, or termination. NIH may appoint a “medical monitor” instead of a DSMB for minimal-risk studies.

### Overall Committee Operations

**Fig. 2.**
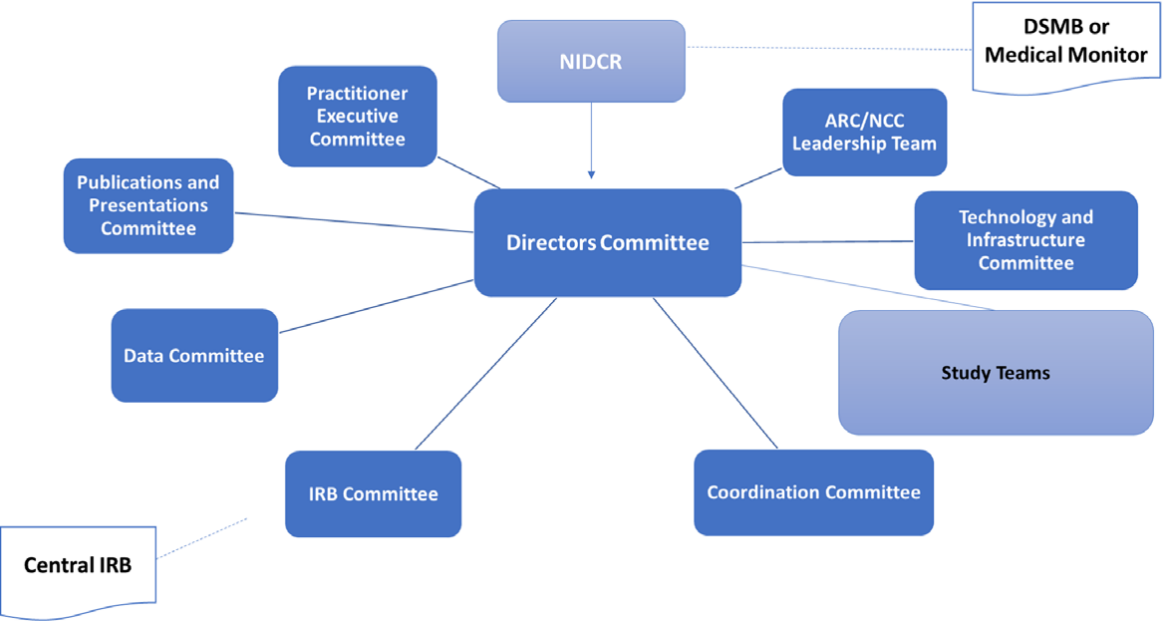
**Network committee structure.** ARC: Administrative and Resource Center; NCC: Network Coordinating Center; DSMB: Data Safety and Monitoring Board; IRB: Institutional Review Board; NIDCR: National Institute of Dental and Craniofacial Research.

Committees manage the bulk of Network operations. The main committee operational structure is depicted in Fig. 2. All committees have both ARC and NCC representation, and one of these two entities takes main responsibility for each committee. Study Principal Investigators lead “Study Teams,” which also include assigned ARC and NCC staff. Committees and Study Teams meet either weekly, biweekly, monthly, or quarterly, depending on the committee.

The Directors Committee provides primary operational oversight. This committee is responsible for optimizing and monitoring overall Network performance, prioritizing tasks, and approving study administration policies and procedures. It also reviews study coordination across nodes and makes decisions about practitioner recruitment, training, and engagement.

The ARC/NCC Leadership Team acts on behalf of the Directors Committee to facilitate its work. This group manages on a weekly to biweekly basis the operational and business components of the Network.

The Coordination Committee discusses node coordination and study implementation issues, shares best practices, and provides a forum for node coordinators and NCC Study Managers to collaborate throughout study development/implementation.

The IRB Committee implements the policies and operations of the CIRB. Because the CIRB has been in operation for several years, it currently meets on a rare, ad hoc basis only.

The Publications and Presentations Committee implements and ensures compliance with the Network’s publications policies document [20].

The Data Committee develops and implements strategies to standardize, collect, manage, analyze and share Network data; provides guidance on data collection tools and prioritizes data quality measures.

The Technology and Infrastructure Committee identifies, prioritizes, and evaluates Network “Hub” needs and functionality.

Study Teams are responsible for developing study-specific documents and procedures to ensure efficient implementation in the Network, providing input regarding data management systems for data collection and quality management activities, preparing documents for CIRB submission, and adhering to NIH policies. During the implementation phase, study teams are responsible for meeting enrollment and retention targets, implementing quality management processes, reporting, and responding to requests regarding study oversight.

### Other Operations

Other key operations of the Network have remained very similar to that which was reported in our earlier publication [9]. These operations include recruitment and retention activities, enrollment processes, practitioner engagement activities, and the benefits of participating as communicated by network practitioners. Previous publications have reported the number and characteristics of Network practitioners, and these numbers also are almost always reported during study-specific publications. Appendix A provides descriptive information about currently enrolled numbers.

### Measures of Study Characteristics

A broad range of study types is conducted in the Network. These include national clinical observational studies, national experimental (randomized clinical trial) studies, national questionnaire studies, pilot clinical studies, clinical simulation studies, and qualitative studies. Most studies are conducted nation-wide, but some involve only one to three regions. Many different funding mechanisms have been used since the Network’s inception in 2005, but since 2019 the Network has conducted only investigator-initiated studies; these are usually funded through specific NIH mechanisms [21,22]. Study length has ranged from less than a year to 3 years.

Clinical topics were categorized into one or more treatment classifications based on the American Dental Association Current Dental Terminology codes [23], with these classifications: Diagnostic; Preventive; Restorative; Endodontics; Periodontics; Prosthodontics (combined categories of Removable Prosthodontics, Maxillofacial Prosthetics, Implant Services, and Fixed Prosthodontics); Oral and Maxillofacial Surgery; Orthodontics; Adjunctive General Services. Clinical topics based on treatment classification were chosen instead of a diagnostic classification system because a single treatment may be the result of different diagnoses, and because in our experience Network clinicians often conceptualize clinical topics in treatment terms. We acknowledge that diagnostic codes, if they were widely recorded in everyday clinical dental practice, would enable a better linkage to health outcomes [24]. The Network also has had an impact internationally by either advising about the formation of new networks in other countries, such as in Japan and Brazil, or collaborating in research studies with investigators in the United Kingdom.

### Measures of Scientific Productivity

Given that a key aspect of the Network’s mission is to conduct research studies and impact the field of clinical research and clinical care, we quantified metrics that have to do with study characteristics and publications: (1) number of studies conducted; (2) breadth of clinical topics investigated; (3) number of practitioners and patients enrolled; (4) number of peer-reviewed scientific publications produced; (5) scientific influence as measured by number of citations, RCRs, and weighted RCRs for Network peer-reviewed publications with a publication date of 2020 or earlier (*n* = 167); and (6) number of different journal titles.

All Network publications are included in the NIH PubMed database in compliance with NIH policy, making access to their PubMed identification number (“PMID”) publicly available and easily entered into the NIH iCite website for quick calculation. We used the iCite tool [12] for citation, RCR, and weighted RCR calculations.

RCR represents the field-normalized and time-normalized citation rate. Article citation rates are divided by an expected citation rate derived from the performance of articles in the same field and benchmarked to a peer comparison group. Fields are defined for each article by using its co-citation network. The RCR is benchmarked to 1.0 for a typical (median) NIH paper in the corresponding year of publication, ensuring that a paper with a median RCR of 1.0 has received the same number of citations per year as the median NIH-funded paper in its field, while a paper with a RCR of 2.0 has received twice as many citations per year as the median NIH-funded paper in its field. RCR data are available for articles that are at least one calendar year old. The database contains many papers that were not NIH funded and the same RCR value translates to a lower percentile ranking for papers that are not NIH funded. The RCR methodology was validated using citation data from about 90,000 published papers emanating from NIH-funded research and comparing calculated RCRs to the opinions on manuscript reach of recognized experts in selected fields. The weighted RCR is the sum of RCRs for Network articles, which weights the article count by their influence relative to NIH-funded papers. A highly influential set of articles will have a higher weighted RCR than the number of total publications, while a set of articles with below-average influence will have a lower weighted RCR than the number of total publications.

## Results

### Study Characteristics

Table 1 lists the characteristics of Network studies completed in data collection or in development. Fifty-six studies have been completed or are in development.

**Table 1.** Characteristics of 58 Network studies completed or in development as of June 2022

Study #	Study title	Study design	# practitioners	# patients	Study type(s)	Clinical topic category(ies)
**Conducted from 2005 to 2012**						
1	Dental tobacco control RCT	Randomized clinical trial	190	11,898	Qx and Clinical	Preventive
2	Practice-based root canal treatment effectiveness^1^	Retrospective cohort study	13	84	Clinical	Endodontics
3	Assessment of caries diagnosis and treatment	Cross-sectional (paper Qx to dentists)	494	--	Qx	Diagnostic; Restorative
4	Reasons for placing the first restoration on permanent tooth surfaces	Cross-sectional; consecutive patients	192	4844	Clinical	Restorative
5	Reasons for replacement or repair of dental restorations	Cross-sectional; consecutive patients	157	6092	Clinical	Restorative
6	CONDOR case-control study of ONJ^2^	Case-control study	81	764	Clinical	Oral/Max Surgery
7	Retrospective cohort study of ONJ	Retrospective cohort study using electronic data	--	572,606	Clinical	Oral/Max Surgery
8	Longitudinal study of dental restorations placed on previously un-restored surfaces	Prospective cohort study	192	4844	Clinical	Restorative
9	Longitudinal study of repaired or replaced dental restorations	Prospective cohort study	157	6092	Clinical	Restorative
10	Development of a patient-based provider intervention for early caries management^1^	Cross-sectional; clinical data collection and Qx	10	336	Qx and Clinical	Diagnostic; Restorative
11	Patient satisfaction with dental restorations	Cross-sectional	159	4680	Clinical	Restorative
12	Prevalence of questionable occlusal caries lesions	Cross-sectional	58	4478	Clinical	Diagnostic; Restorative
13	Longitudinal study of questionable occlusal caries lesions	Prospective cohort study	58	4478	Clinical	Diagnostic; Restorative
14	Hygienists’ internet tobacco cessation RCT	Randomized clinical trial	100	1814	Clinical	Preventive
15	Blood glucose testing in dental practice	Cross-sectional	23	387	Clinical	Diagnostic
16	Assessing the impact of participation in PBRNs on patient care	Cross-sectional (paper Qx with dentists and dental hygienists)	613	--	Qx	Adjunctive
17	Assessing the impact of participation in PBRNs on patient care - repeated 2 years	Cross-sectional (paper Qx with dentists and dental hygienists)	556	--	Qx	Adjunctive
18	Peri-operative pain and root canal therapy	Prospective cohort study	55	655	Clinical	Endodontics
19	Persistent pain and root canal therapy	Prospective cohort study	55	655	Clinical	Endodontics
20	Diagnoses for persistent dentoalveolar pain following root canal therapy^1^	Nested case series study	63	354	Clinical	Endodontics
21	Primary care management of TMD	Cross-sectional (electronic Qx with dentists)	434	--	Qx	Diagnostic; Oral/Max Surgery; Prosthodontics
22	Infrastructure update survey	Cross-sectional (electronic Qx with dentists)	649	--	Qx	Adjunctive
23	Isolation techniques used when performing root canal treatment	Cross-sectional (electronic and paper Qx with dentists)	1491	--	Qx	Endodontics
24	Management of suspicious occlusal caries lesions	Randomized clinical trial	125	3093	Qx and Clinical	Diagnostic; Restorative
25	Management of dentin hypersensitivity (two parts)	#1: Cross-sectional (electronic Qx with dentists)	#1, #2: 200	#2: 1876	Qx and Clinical	Restorative
		#2: Prospective cohort study				
26	Reducing prescription opioid misuse	Cross-sectional (electronic Qx with dentists)	822	--	Qx	Adjunctive
27	Understanding dental information networks	Cross-sectional (electronic Qx with dentists)	1860	--	Qx	Adjunctive
28	Quit Advisor DDS smoking cessation study^1^	Feasibility non-randomized controlled clinical trial	30	248	Clinical	Preventive
29	Factors for Successful Crowns Questionnaire	Cross-sectional (electronic Qx with dentists)	1852	--	Qx	Prosthodontics
30	Factors for Successful Crowns Clinical Study	Prospective cohort study	207	3847	Clinical	Prosthodontics
31	Leveraging EDR data for clinical research	Retrospective cohort study (EDR extraction of patients who received root canal treatment and select restorations)	99	217,887	Clinical	Endodontics; Restorative
32	Common practices of head & neck examinations in U.S. dental offices	Cross-sectional (electronic and paper Qx with dentists)	1126	--	Qx	Diagnostic
33	Cracked tooth registry study	Prospective cohort study	236	3017	Clinical	Diagnostic; Restorative
34	Risk for oral cancer/HPV study	Prospective cohort study	37	1025	Clinical	Diagnostic
35	Anterior open-bite treatment	Prospective cohort study	96	358	Clinical	Orthodontics
36	Predicting root canal treatment outcomes	Prospective cohort study	172	1883	Clinical	Endodontics
37	TMD treatment methods	Prospective cohort study	185	1901	Clinical	Diagnostic; Oral/Max Surgery; Prosthodontics
38	Multi-risk assessment in the dental office (two parts)^1^	#1: Cross-sectional (paper Qx with dentists); #2: Cross-sectional clinical study	#1: 475 #2: 30	#2: 857	#1: Qx; #2: Clinical	Diagnostic
39	Prophylactic use of antibiotics in dental office	Cross-sectional (electronic and paper Qx with dentists)	2169	--	Qx	Adjunctive
**Conducted from 2020 to present**						
40	Assessment of PPE for dental healthcare professionals^1^	Prospective cohort study	70	--	Clinical	Adjunctive
41	COVID-19 Research Registry	Cross-sectional (electronic Qx with dentists)	1819	--	Qx	Adjunctive
42	Evaluation of aerosol-generating procedures in dental settings^1^	Clinical simulation	--	--	Clinical simulation	Adjunctive
43	Evaluation of aerosol composition in dental settings^1^	Clinical simulation	--	--	Clinical simulation	Adjunctive
44	Particle topography and aerosol size distribution in dental settings in the COVID-10 era^1^	Clinical simulation	--	--	Clinical simulation	Adjunctive
45	Selective versus nonselective caries removal in permanent teeth	Cross-sectional (electronic Qx with dentists)	478	--	Qx	Restorative
46	Treatment of patients on conventional and direct oral anticoagulants	Cross-sectional (electronic Qx with dentists)	866	--	Qx	Adjunctive
47	Coronavirus vaccine acceptability and readiness among dentists	Cross-sectional (electronic Qx with dentists)	550	--	Qx	Adjunctive
48	Pragmatic return to effective dental infection control through triage and testing^1^	Prospective cohort study	30	43	Clinical	Adjunctive
49	Dental management of patient with special health care needs	Cross-sectional (electronic Qx with dentists)	505	--	Qx	Adjunctive
50	An innovative mDentistry eHygiene study amid the COVID-19 pandemic^1^	Prospective cohort feasibility study	18	62	Clinical	Adjunctive
51	Acute pain pathways^4^	Prospective cohort study	In progress; 25 planned	In progress; 150 planned	Clinical	Adjunctive
52	Understanding pain after dental procedures	Prospective cohort study	In progress; 150 planned	In progress; 3000 planned	Clinical	Endodontics; Periodontics; Oral/Max Surgery
53	Dental implant registry	Prospective cohort study	200 (planned)	1000 (planned)	Clinical	Periodontics; Prosthodontics; Oral/Max Surgery
54	Diagnosis of dental hard tissue conditions^3^	Cross-sectional (electronic Qx with dentists and patients)	660 (planned)	1350 (planned)	Qx	Diagnostic
55	Effectiveness of nicotine replacement sampling in dental practices^2^	Randomized clinical trial	50 (planned)	1200 (planned)	Clinical	Preventive
56	Mental health screening and referral in dental practices^1^	Cross-sectional	60 (planned)	150 (planned)	Clinical	Diagnostic
57	Risk factors associated with the prevalence of peri-implantitis disease	Cross-sectional	100 (planned)	1000 (planned)	Clinical	Periodontics; Prosthodontics; Oral/Max Surgery
58	Indications for periodontal adjunctive antibiotics in dental practice	Randomized clinical trial	35 (planned)	1050 (planned)	Clinical	Periodontics;

1 One network region only

2 Two network regions only

3 Three network regions only

4 One network region only; the study is a collaboration with five other sites at academic health center (i.e., non-PBRN) clinics in the USA, funded by U01-FD-005938. The planned enrollment of 1500 patients across the entire collaboration is at 1235 as of June 9, 2022.

Abbreviations: EDR: electronic dental record; HPV: human papillomavirus; ONJ: osteonecrosis of the jaw; Qx: questionnaire; PPE: personal protective equipment; RCT: randomized clinical trial; TMD: temporomandibular disorders

Detailed study information can be accessed at http://nationaldentalpbrn.org/studies.php

An early version of this table was published in 2018 [17].

Of the studies that have completed enrollment (studies 1–50), 19,827 practitioners have been enrolled (some participated in multiple studies), along with 70,665 patient participants (excluding data-only studies). Two data-only studies (studies 7 and 31) examined electronic health records for 790,493 patients.

The 58 studies listed in Table 1 comprise 22 questionnaire studies, 38 clinical studies, and 3 clinical simulation studies. Study designs have included 28 cross-sectional designs, 1 case-control design, 1 nested case series design, 18 prospective cohort studies, 3 retrospective cohort studies, 1 non-randomized feasibility controlled clinical trial, 5 randomized controlled clinical trials, and 3 clinical simulations.

Clinical topics were categorized as investigating 14 Diagnostic topics, 4 Preventive topics, 14 Restorative topics, 8 Endodontics topics, 4 Periodontics topics, 6 Prosthodontics topics, 7 Oral and Maxillofacial Surgery topics, 1 Orthodontics topic, and 17 Adjunctive General Services topics.

### Publication Productivity

The Network has produced 193 peer-reviewed publications to date [20]. A total of 167 had publication dates of 2020 or earlier. Peer-reviewed publications have appeared in a total of 62 different journal titles so far, which comprise a broad range of scientific disciplines.

**Fig. 3.**
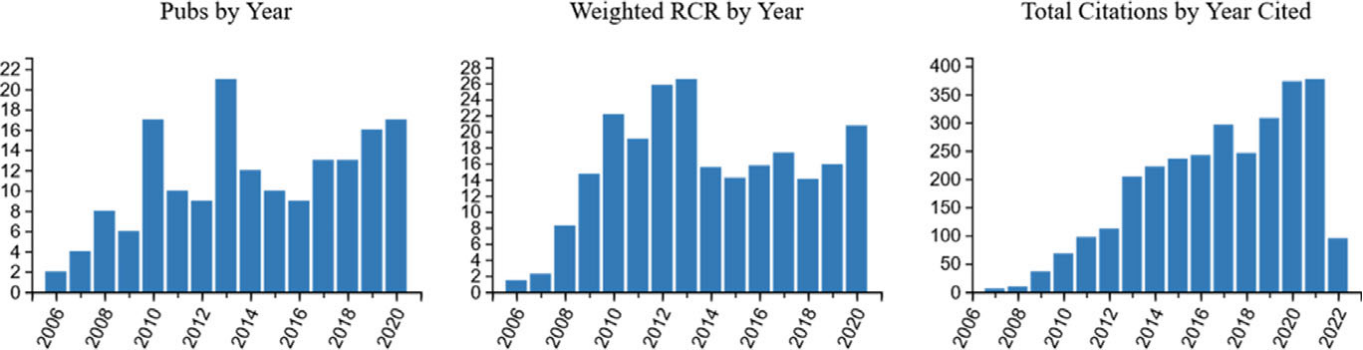
**Graphic presentation of iCite results from Network publications that were published 2006–2020 (*n* = 167), as of March 29, 2022.** The graphs were produced by the iCite system [12]. RCR: Relative Citation Ratio.

Fig. 3 shows results from the iCite analysis of the 167 publications that had publication dates of 2020 or earlier, displaying number per year, weighted RCR by year, and total citations by year cited. Not shown in the figure are these metrics: 11.13 publications per year; 2927 total citations; a mean (SE) of 17.53 (1.79) and a median of 11.0 citations per publication; mean (SE) of 2.09 (0.17) citations per publication per year. The mean (SE) RCR was 1.40 (0.11), the median was 1.07, and the weighted RCR was 233.84.

We compared the percentile ranking of the 167 publications to the corpus of PubMed publications based on RCRs; nine of the 167 publications were ranked above the 90^th^ percentile and 15 articles were ranked between the 80^th^ and 90^th^ percentiles. An article-specific report is publicly available [25].

The weighted RCR (234 in the case of this analysis) was considerably higher than the number of total publications (167 in the case of this analysis), which indicated that this set of publications was highly influential relative to the average paper in the PubMed database.

## Discussion

Having described the Network’s organizational changes in 2019 in the context of a Network history that began in 2005, we have documented that a PBRN can productively engage community practitioners, patients, and clinical research investigators over a sustained multi-year period. This adds to the evidence and conclusions made in a recent review of PBRNs [8]. Our report focuses on one particular network to document its productivity as measured by recruiting and engaging practitioners in research, completing many studies, and disseminating research findings through peer-reviewed publications. These results suggest that everyday practitioners can be crucial partners in developing, implementing, and disseminating scientific research, and that community practices can be an effective venue for clinical research. Network studies have generated numerous, timely, and influential publications, in a broad range of clinical topic areas.

That the median RCR of Network publications exceeded 1.0 and the weighted RCR substantially exceeded the total number of publications upon which the analysis was based, demonstrate that the network’s publications have had a greater-than-average influence in their fields [11]. Because of its mission, the Network attempts to balance its interest in communicating with and disseminating to a clinical audience, with its desire to maintain a strong scientific credibility. Consequently, the Network decides on many occasions to target a particular peer-reviewed scientific journal because it has a heavily clinical readership, even though the journal’s impact factor is not as high as other target journals that we believe would accept the manuscript. Consequently, our *a priori* goal before conducting the iCite analysis was much lower (25^th^ percentile) than the actual percentile obtained (higher than the 50^th^ percentile). As one of several methods to quantify publication quality or its impact on the field, the RCR has performed better than journal impact factor, citations per year, or the Thompson–Reuters ratio [11,26,27]. Establishing consensus about methods to quantify success and productivity of large research groups may be important to justify continued funding or establishing new networks [28,29]. Self-citation and publication frequency among network coauthors has the potential to inflate RCRs, but the effect is minimized by the wide range of study topics that limit the extent of self-citation (numerator), while articles overly benefitting from self-citation would increase the field citation rate (denominator). A limitation to PBRN research is that a high RCR demonstrates prominence among scientific peers, yet does not capture the Network’s influence on everyday clinical practice and patient health, which is the ultimate goal of the Network. Impact on everyday practice can be the key research question for some studies [30-32], although the typical goal is to contribute to the evidence base in a manner similar to individual “R01” studies or clinical trials. PBRNs offer recruitment sites (community practices) that complement or supplement academic health center sites with geographic and patient demographic diversity, while also providing an infrastructure to engage simultaneously both academic clinical scientists and the “end-users” (community practitioners) of results from the studies, at each step of the study development and implementation process [9,32].

The National Dental PBRN has operated at a high level of scientific productivity and has demonstrated the feasibility and effectiveness of PBRNs as a research context. The network seeks to foster a future in which research and quality improvement are done routinely in everyday clinical practice – just because that is what health care providers do as a profession. The ultimate goal is to advance the delivery of evidence-based care into daily clinical practice for the benefit of patients.

## Supplementary material

For supplementary material accompanying this paper visit https://doi.org/10.1017/cts.2022.421. click here to view supplementary material
