# Supplementary material for: Structure, function, and productivity from the National Dental Practice-Based Research Network
Source: J Clin Transl Sci. 2022 Jun 22;6(1):e87. doi: 10.1017/cts.2022.421 (PMC9379935; doi:10.1017/cts.2022.421)
Supplement: Supplementary file 1 [file S2059866122004216sup001.doc]

# Appendix A

**Practitioner Characteristics**

**By Node and Overall ***

|  | **Western** | **Midwest** | **Southwest** | **South**  **Central** | **South**  **Atlantic** | **Northeast** | **International** | **Overall** |
| --- | --- | --- | --- | --- | --- | --- | --- | --- |

| **Count of Practitioners** | 1113 | 1102 | 1452 | 1459 | 1315 | 1270 | 139 | 7850 |
| --- | --- | --- | --- | --- | --- | --- | --- | --- |

**Sex**

| Male | 552 (49.6%) | 457 (41.5%) | 697 (48.0%) | 711 (48.7%) | 510 (38.8%) | 736 (58.0%) | 81 (58.3%) | 3744 (47.7%) |
| --- | --- | --- | --- | --- | --- | --- | --- | --- |
| Female | 551 (49.5%) | 631 (57.3%) | 737 (50.8%) | 733 (50.2%) | 795 (60.5%) | 519 (40.9%) | 58 (41.7%) | 4024 (51.3%) |
| Unknown/Missing | 10 (0.9%) | 14 (1.3%) | 18 (1.2%) | 15 (1.0%) | 10 (0.8%) | 15 (1.2%) | 0 (0.0%) | 82 (1.0%) |

**Age**

| Age (Mean) | 50.7 | 46.9 | 51.4 | 49.2 | 48.8 | 54.6 | 44.4 | 50.2 |
| --- | --- | --- | --- | --- | --- | --- | --- | --- |
| Age Range | (24‐95) | (22‐93) | (16‐90) | (20‐90) | (21‐87) | (16‐95) | (20‐80) | (16‐95) |

**Ethnicity**

| Of Hispanic origin | 62 (5.6%) | 48 (4.4%) | 193 (13.3%) | 34 (2.3%) | 187 (14.2%) | 68 (5.4%) | 14 (10.1%) | 606 (7.7%) |
| --- | --- | --- | --- | --- | --- | --- | --- | --- |
| Not of Hispanic or Latino origin | 1030 (92.5%) | 1029 (93.4%) | 1219 (84.0%) | 1405 (96.3%) | 1109 (84.3%) | 1179 (92.8%) | 122 (87.8%) | 7093 (90.4%) |
| Prefer not to answer/Missing | 21 (1.9%) | 25 (2.3%) | 40 (2.8%) | 20 (1.4%) | 19 (1.4%) | 23 (1.8%) | 3 (2.2%) | 151 (1.9%) |

**Race**

| American Indian or Alaska Native | 7 (0.6%) | 3 (0.3%) | 9 (0.6%) | 4 (0.3%) | 3 (0.2%) | 0 (0.0%) | 0 (0.0%) | 26 (0.3%) |
| --- | --- | --- | --- | --- | --- | --- | --- | --- |
| Asian | 191 (17.2%) | 99 (9.0%) | 177 (12.2%) | 57 (3.9%) | 88 (6.7%) | 108 (8.5%) | 35 (25.2%) | 755 (9.6%) |
| Native Hawaiian or Other Pacific Islander | 5 (0.4%) | 4 (0.4%) | 2 (0.1%) | 0 (0.0%) | 2 (0.2%) | 0 (0.0%) | 0 (0.0%) | 13 (0.2%) |
| Black or African‐American | 21 (1.9%) | 25 (2.3%) | 64 (4.4%) | 139 (9.5%) | 78 (5.9%) | 47 (3.7%) | 2 (1.4%) | 376 (4.8%) |
| White or Caucasian | 776 (69.7%) | 884 (80.2%) | 1021 (70.3%) | 1199 (82.2%) | 1037 (78.9%) | 1000 (78.7%) | 70 (50.4%) | 5987 (76.3%) |
| Asian Indian/East Indian | 16 (1.4%) | 23 (2.1%) | 42 (2.9%) | 7 (0.5%) | 14 (1.1%) | 30 (2.4%) | 5 (3.6%) | 137 (1.7%) |
| Middle Eastern | 10 (0.9%) | 14 (1.3%) | 27 (1.9%) | 7 (0.5%) | 18 (1.4%) | 21 (1.7%) | 11 (7.9%) | 108 (1.4%) |
| Other | 28 (2.5%) | 5 (0.5%) | 35 (2.4%) | 13 (0.9%) | 24 (1.8%) | 14 (1.1%) | 5 (3.6%) | 124 (1.6%) |
| More Than One Race | 32 (2.9%) | 17 (1.5%) | 24 (1.7%) | 15 (1.0%) | 21 (1.6%) | 21 (1.7%) | 7 (5.0%) | 137 (1.7%) |
| Prefer not to answer | 27 (2.4%) | 28 (2.5%) | 51 (3.5%) | 18 (1.2%) | 30 (2.3%) | 29 (2.3%) | 4 (2.9%) | 187 (2.4%) |

**Primary Occupation**

| Dentist Practicing within the U.S. | 758 (68.1%) | 648 (58.8%) | 1030 (70.9%) | 748 (51.3%) | 647 (49.2%) | 921 (72.5%) | -- | 4752 (60.5%) |
| --- | --- | --- | --- | --- | --- | --- | --- | --- |
| Hygienists/Dental Therapist | 201 (18.1%) | 286 (26.0%) | 268 (18.5%) | 333 (22.8%) | 450 (34.2%) | 154 (12.1%) | -- | 1692 (21.6%) |
| All other | 154 (13.8%) | 168 (15.2%) | 154 (10.6%) | 378 (25.9%) | 218 (16.6%) | 195 (15.4%) | 139 (100.0%) | 1406 (17.9%) |

**Practice Setting**

| Dentist, private practice | 522 (46.9%) | 428 (38.8%) | 824 (56.7%) | 630 (43.2%) | 542 (41.2%) | 753 (59.3%) | -- | 3699 (47.1%) |
| --- | --- | --- | --- | --- | --- | --- | --- | --- |
| Dentist, managed care or corporate | 121 (10.9%) | 91 (8.3%) | 68 (4.7%) | 20 (1.4%) | 20 (1.5%) | 10 (0.8%) | -- | 330 (4.2%) |
| Dentist, school or hospital | 35 (3.1%) | 75 (6.8%) | 58 (4.0%) | 54 (3.7%) | 41 (3.1%) | 105 (8.3%) | -- | 368 (4.7%) |
| Dentist, government (Armed forces, VA, Etc.) | 42 (3.8%) | 24 (2.2%) | 31 (2.1%) | 17 (1.2%) | 23 (1.7%) | 22 (1.7%) | -- | 159 (2.0%) |
| Dentist, Public health, CHC, publicly funded clinic | 38 (3.4%) | 30 (2.7%) | 49 (3.4%) | 27 (1.9%) | 21 (1.6%) | 31 (2.4%) | -- | 196 (2.5%) |
| Dental Therapist/Hygienist | 201 (18.1%) | 286 (26.0%) | 268 (18.5%) | 333 (22.8%) | 450 (34.2%) | 154 (12.1%) | -- | 1692 (21.6%) |
| Students | 14 (1.3%) | 61 (5.5%) | 16 (1.1%) | 224 (15.4%) | 78 (5.9%) | 57 (4.5%) | -- | 450 (5.7%) |
| Dental Assistants/Office Staff | 44 (4.0%) | 16 (1.5%) | 24 (1.7%) | 46 (3.2%) | 14 (1.1%) | 38 (3.0%) | -- | 182 (2.3%) |
| All other | 96 (8.6%) | 91 (8.3%) | 114 (7.9%) | 108 (7.4%) | 126 (9.6%) | 100 (7.9%) | 139 (100.0%) | 774 (9.9%) |

***** As of January 16, 2022
